# Trans-generational Immune Priming in Invertebrates: Current Knowledge and Future Prospects

**DOI:** 10.3389/fimmu.2019.01938

**Published:** 2019-08-14

**Authors:** Guillaume Tetreau, Julien Dhinaut, Benjamin Gourbal, Yannick Moret

**Affiliations:** ^1^Université de Perpignan Via Domitia, IHPE UMR 5244, CNRS, IFREMER, Univ. Montpellier, Perpignan, France; ^2^Université Grenoble Alpes, CNRS, CEA, IBS, Grenoble, France; ^3^UMR CNRS 6282 BioGéoSciences, Équipe Écologie Évolutive, Université Bourgogne-Franche Comté, Dijon, France

**Keywords:** trans-generational immune priming, invertebrate immunity, host-pathogens interaction, ecology and evolution, molecular mechanisms, scenarios

## Abstract

Trans-generational immune priming (TGIP) refers to the transfer of the parental immunological experience to its progeny. This may result in offspring protection from repeated encounters with pathogens that persist across generations. Although extensively studied in vertebrates for over a century, this phenomenon has only been identified 20 years ago in invertebrates. Since then, invertebrate TGIP has been the focus of an increasing interest, with half of studies published during the last few years. TGIP has now been tested in several invertebrate systems using various experimental approaches and measures to study it at both functional and evolutionary levels. However, drawing an overall picture of TGIP from available studies still appears to be a difficult task. Here, we provide a comprehensive review of TGIP in invertebrates with the objective of confronting all the data generated to date to highlight the main features and mechanisms identified in the context of its ecology and evolution. To this purpose, we describe all the articles reporting experimental investigation of TGIP in invertebrates and propose a critical analysis of the experimental procedures performed to study this phenomenon. We then investigate the outcome of TGIP in the offspring and its ecological and evolutionary relevance before reviewing the potential molecular mechanisms identified to date. In the light of this review, we build hypothetical scenarios of the mechanisms through which TGIP might be achieved and propose guidelines for future investigations.

## Introduction

Parasites/pathogens can cause significant damage to host fitness. In response, hosts have evolved a range of defense mechanisms reducing their negative impact (1). These mechanisms include behavioral defenses and physical barriers that help to prevent infection, and the immune system that has evolved to control infection inside hosts. In vertebrates, the efficiency of the immune system relies on a combination of innate and acquired responses and on the ability of recovered hosts to remain protected for an extended period of time (2). Furthermore, an important aspect of the acquired immune response of vertebrates is the production of specific immune effectors, the antibodies. These can be transferred by infected mothers to their offspring via the placenta and milk in mammals, or via the egg yolk in birds, reptiles, and fishes (3). Such a maternal transfer of immunity provides newborns with early protection against prevalent parasites/pathogens while their own immune system becomes mature.

Invertebrates lack the immune machinery responsible for the acquired immune response of vertebrates (4). Their innate immunity mainly depends on germ-line encoded receptors recognizing generic conserved pathogen epitopes. Despite this, cumulative evidences now demonstrate that the innate immune system of invertebrates can produce immune responses involving memory, either non-specific or specific (5, 6). As different mechanisms underlie acquired immunity in vertebrate taxa, the general term “immunological priming” (or immune priming) is currently used to refer to the “adaptive” innate immune response of invertebrates (7). Moreover, although invertebrates lack antibodies that vertebrate females transfer to their offspring, maternal (and paternal) effects on the offspring immunity occur in invertebrates too (5). This is also called “trans-generational immune priming” (TGIP) (8). In invertebrates, TGIP specifically refers to the vertical transmission of the immunological experience from the parent(s) to the offspring, which may also include horizontal transfers between adults and between adults and other parents' offspring (9). Such a transmission of the parental immunological experience may take different forms. Parents could transfer either immune effectors or signals to the offspring that may prepare or stimulate its immune system to deal with the pathogens previously met by the parents. The involvement of each of these processes or both is not known from a functional point of view yet. TGIP currently raises considerable questions related to its mechanisms, its epidemiological impact on disease dynamics and its evolution. Since its characterization two decades ago, invertebrate TGIP has been the focus of an increasing interest. The phenomenon of TGIP has now been tested in several invertebrate systems with the aim to study it at both functional and evolutionary levels. However, drawing an overall picture of TGIP from available studies is a tedious task. The major issues encountered are the lack of clear and consistent evidence for TGIP. This is not only due to the sheer complexity of different pathways and mechanisms that can lead to TGIP, but also because of the biases and inconsistent experimental designs that have been used to assay TGIP. Several attempts to review TGIP have already been made. TGIP was generally a specific part of a more general immunological review (4, 10–14) and only few dedicated reviews were published (9, 15, 16). Their objective was to provide a global overview rather than an in-depth systematic and extensive review of all aspects of TGIP.

The present review therefore aims to confront all the data published to date in order to establish a theoretical and practical framework for helping in the experimental design and data analysis of future studies on TGIP. The novelty of our review relies on the unprecedented combination of an in-depth analysis of the ecological and evolutionary features of TGIP with a comprehensive and critical investigation of the molecular mechanisms of TGIP identified and/or suspected. To this purpose, after a description of all the articles reporting experimental investigation of TGIP in invertebrates, we propose a critical analysis of the experimental procedures performed to study TGIP. From the most recent advances on TGIP, we also examine whether this aspect of invertebrate immunity could be adaptive from selective pressures by repeated parasite/pathogen infections, and consider the ecological conditions that may affect its evolution and shape its characteristics. Finally, we review the different potential molecular mechanisms identified to date, build hypothetical scenarios of the mechanisms leading to TGIP based on empirical data and propose guidelines for future investigations.

### Occurrence of TGIP in the Tree of Life

**Figure 1:**
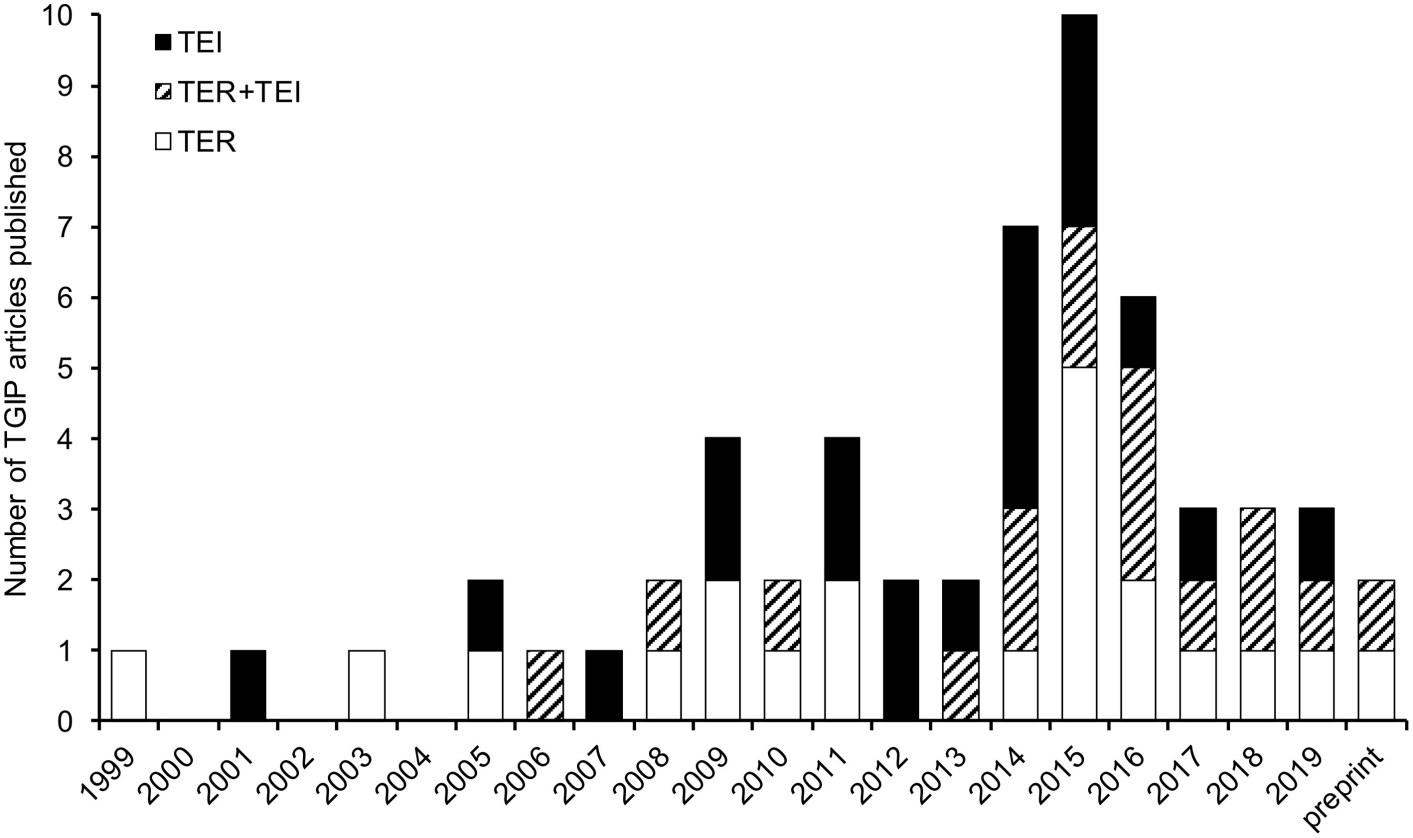
All the 57 articles published in peer-reviewed journals that investigated TGIP in invertebrates. Articles quantifying the consequence of parental pathogen exposure on the outcome of infection in offspring (e.g., parasite prevalence and intensity, host fitness, and survival) are indicated as TER (trans-generational effect on resistance; gray color). Articles focusing on the impact on offspring immunity (e.g., number of hemocytes, modified expression or activity of AMPs, PPO, or immune pathways) are indicated as TEI (trans-generational effect on immunity; black color), following the updated nomenclature proposed by Pigeault et al. (19). Articles that evaluated both parameters are hatched in black and gray and indicated as TER+TEI. All information relative to the 57 TGIP articles published to date are available in Supplementary Table 1. Considering that the term TGIP is not used by all authors and that some investigated it without highlighting it clearly in the title and/or abstract, complementary searches were performed to retrieve all TGIP articles. Different search engines were used for identifying peer-reviewed (PubMed, Web of Science, Google Scholar, Biological Abstracts) and preprint articles (bioRxiv). We used different combination of keywords including notably “transgenerational immune priming,” “immune priming across generations,” “multigenerational immunity,” “vertical transfer immunity,” “maternal/parental transfer immunity,” “maternal/parental effect immunity,” “transfer immune memory,” “offspring immunity invertebrate,” “offspring immunity insect.” In addition, several articles dealing with within-generation invertebrate immune priming and immune memory were investigated for evidence of experimental design and results that relate to TGIP.

**Figure 2:**
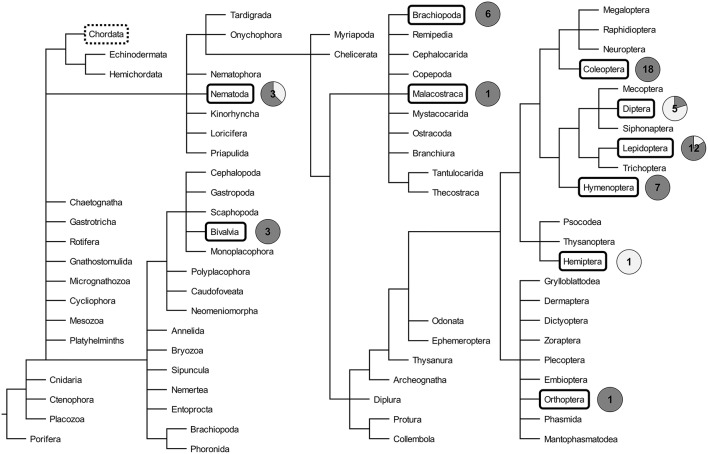
Phylogeny of invertebrates, adapted from Tree of Life Web Project (20). Taxa in which transgenerational immune priming has been investigated are boxed. The circle charts indicate the proportion of TGIP studies that reported the presence (dark gray) and the absence (light gray) of TGIP for each phylogenetic group boxed. The number indicated inside the circle chart is the number of TGIP studies reported to date for each phylogenetic group. The group of Chordata, which includes the vertebrates, is highlighted by a dotted box.

The existence of TGIP was already hypothesized in the early 1900s (17). The first empirical evidence of TGIP was provided in 1999 in the crustacean *P. monodon*. Mothers exposed to β-glucans induced protection of offspring against the white spot syndrome associated virus (WSSV) (18). Since then, a total of 57 articles investigating TGIP in invertebrates has been published (Figure 1). The details of each of these articles can be found in the Supplementary Table 1. The number of articles published on this topic remained low for more than a decade and then increased, with half of the articles published during the last 5 years, reflecting the recent interest for this new field of research in invertebrate immunity (Figure 1). Interestingly, a drop in the total number of articles published since 2017 has been observed. This sudden pattern of publication dynamics does not reflect that every aspect of invertebrate TGIP is known. Instead, it may indicate how difficult it is now to propose real groundbreaking progress on the understanding of this phenomenon. Indeed, the majority of the studies published until 2017 mostly reported the phenomenal occurrence of TGIP in invertebrates. Very few of them attempted to figure out a comprehensive description of its functional processes and/or its evolution, which is a much more difficult and time-consuming task. Since 2017, articles tend to be more comprehensive and investigate the details of the mechanisms and evolutionary ecology of TGIP. So far, TGIP has been investigated on 25 different invertebrate species (Figure 2; Supplementary Table 1). TGIP studies are strongly biased toward arthropods, representing ~90% of all TGIP articles, and many groups have not been investigated yet (Figure 2).

While TGIP has been evidenced in all coleopteran, crustacean, hymenopteran, orthopteran, and mollusk species investigated to date, some other phylogenetic groups exhibit more contrasted patterns (Figure 2). Indeed, only one out of the five articles on Diptera provided evidence for TGIP (Supplementary Table 1). TGIP was found in *Anopheles gambiae* larvae to the microsporidia *Vavraia culicis* (21), whereas exposure of *Drosophila melanogaster* mothers to bacteria, and exposure of three mosquito species to *Plasmodium sp*. and to negatively-charged beads did not trigger any increased immune protection of the offspring (22–25). Similarly, for Lepidoptera, two articles focusing on *Plodia interpunctella* exposed to the bacteria *B. thuringiensis* and on *Trichoplusia ni* challenged with *Autographa californica multiple nucleopolyhedrovirus* did not find evidence of TGIP (26, 27). Interestingly, parental challenge of the same lepidopteran species to different pathogens (i.e., *P. interpunctella* to the *granulosis virus (PiGV)* and *T. ni* to the bacteria *Escherichia coli* and *Micrococcus luteus*) provided an immune protection of the offspring (28–30). This suggests that TGIP might depend on the pathogen used for priming and/or on the procedure used for infection (discussed in detail afterwards), without excluding additional effects such as the host genotype.

The current overview of the presence/absence of TGIP in the tree of life must be considered with caution for two main reasons. First, there is a high heterogeneity in the TGIP articles published, both in term of experimental approaches and in their reliability. While some studies provide reliable data, others suffer from biases that can affect the outcome of the experiments and question whether their results really demonstrate the presence of TGIP. This includes pseudoreplication in the experimental design, non-adequate statistical tests, and/or low statistical power due to small sample sizes (risk of type 1 errors), potential direct transmission of the pathogen, etc. (Supplementary Table 1). This phenomenon is widespread notably in many case reports published before 2017 as stated above. It, however, tends to disappear with the establishment of TGIP as a standalone field of research with few recognized teams of scientists aiming at publishing more comprehensive and detailed studies. Second, TGIP studies are biased toward model species and positive results, with only 12.2% of studies reporting an absence of TGIP. This bias can be explained by the higher difficulty to publish negative than positive results, providing a distorted view of the occurrence of TGIP in invertebrates (31). In order to provide a representative overview of TGIP in the tree of life, additional relevant host/pathogen combinations, including neglected non-model species, should be studied. However, absence of evidence does not always mean evidence of absence for TGIP. Studies might just lack statistical power to demonstrate the absence of TGIP and/or miss the conditions in which TGIP occurs. Special care should be given to the experimental design, the adequate infection procedure to both the pathogen and the host, and the replication procedure to ensure that proper statistics can be conducted to demonstrate the presence or absence of TGIP in each case. These points are discussed in the following parts and guidelines are provided to help in the design of future studies.

## Critical Analysis of Approaches Used to Study TGIP

The different studies exhibited a high variation in the procedure that they used to investigate TGIP. This could influence the outcome of the experiments and whether the presence (or absence) of TGIP reported is biologically relevant (Table 1; Supplementary Table 1). Most notably, we identified three major parameters that showed high variability between studies and that we believe are key to compare the results obtained from different groups of scientists, between different pathogens from the same study, and to properly discuss the relevance of the results obtained: the infection procedure, the sex of the insect host and the developmental stage studied. The influence of some of these parameters has already been discussed in the context of immune priming and within-generation immunological memory in invertebrates (5). Here, we focus on the trans-generational consequences.

**Table 1:** Summary of the main features of TGIP identified in different phylogenetic groups and species.

**Group studied**	**Species studied**	**Parental priming**	**Priming way**	**TER benefit**	**TEI benefit**	**TGIP Costs**	**References**
Coleoptera	*Anoplophora glabripennis*	Bacteria^†^ and fungi°^†^	Injected	Survival (adults)	Not tested	Not tested	(32)
	*Rhynchophorus ferrugineus*	Bacteria°	Injected	Not tested	Enhanced PO and antibacterial activity (larvae)	Not tested	(33)
	*Tenebrio molitor*	Bacteria^†^, fungi^†^ or LPS	Injected	Survival (adults)	Enhanced antibacterial activity (larvae). Enhanced antimicrobial activity (eggs). Enhanced hemocyte concentration or PO activity (adults)	Trade-off between maternal immune response and egg protection (antibacterial activity). Longer offspring development time.	(8, 34–40)
	*Tribolium castaneum*	Bacteria°^†^ and/or parasite°	Injected, ingested or parasitized	Survival (adults). Modified bacterial density dynamics.	Modified gene expression (eggs, larvae). Enhanced expression of PGRP receptors and enhanced PO activity (adults)	Lower antibacterial activity in adults. Longer developmental time. Lower offspring fecundity	(41–47)
Crustacea	*Artemia sp*.	Bacteria°	Ingested	Survival (larvae)	Enhanced gene expression (larvae)	Not tested	(48, 49)
	*Daphnia magna*	Bacteria°	Ingested	Lower susceptibility (larvae)	Not tested	Not tested	(50, 51)
	*Penaeus monodon*	b-1,3-1,6-glucan	Injected and ingested	Survival (larvae)	Not tested	Not tested	(18)
Diptera	*Anopheles gambiae*	Microsporidia°	Ingested	Lower susceptibility (adults)	Not tested	Longer offspring developmental time	(21)
Hemiptera	*Myzus persicae*	Parasitoid°	Parasitized	Lower susceptibility (nymphs)	Not tested	Not tested	(52)
Hymenoptera	*Apis mellifera*	Bacteria^†^	Injected	Survival (larvae)	Enhanced prohemocytes-to-hemocytes differentiation (larvae)	Not tested	(53)
	*Bombus terrestris*	Bacteria^†^ or LPS	Injected	Not tested	Enhanced antibacterial activity (worker adults, eggs). Enhanced PO activity (male adults). Enhanced gene expression (worker adults)	Parents produced less offspring. Decreased PO in offspring adults workers. Increased susceptibility in adults to a parasite unrelated to the maternal challenge	(54–58)
	*Crematogaster scutellaris*	Fungi°^†^	Contact	Survival (larvae)	Not tested	Not tested	(59)
Lepidoptera	*Galleria mellonella*	Bacteria°	Ingested	Not tested	Modified gene expression (eggs)	Not tested	(60)
	*Manduca sexta*	Peptidoglycan, bacteria°^†^	Injected	Reduce parasitoid development and emergence (eggs). Faster infection clearance	Enhanced PO and antibacterial activity (eggs, larvae). Enhanced gene expression (eggs, larvae). Decreased DNA methylation. Increased histone acetylation	Faster reduction of antibacterial activity in adult offspring. Reduced offspring fecundity. Longer larval development	(61–65)
	*Plodia interpunctella*	Virus° (not efficient with bacteria° and fungi°)	Ingested	Lower susceptibility (adults)	Not tested	Not tested	(29)
	*Trichoplusia ni*	Bacteria° (not efficient with virus°)	Ingested	No (but just tested with one virus)	Enhanced PO activity (larvae). Modified gene expression (eggs, larvae)	Not tested	(28, 66)
Mollusca	*Chlamys farreri*	Bacteria^†^	Injected	Survival (all stages)	Increased number of proteins and mRNA (eggs, larvae, and adults)	Not tested	(67)
	*Crassostrea gigas*	Poly(I:C)	Injected	Survival (larvae)	Modified gene expression (larvae)	Not tested	(68, 69)
Nematoda	*Caenorhabditis elegans*	Virus°	Ingested	Not tested	Parental RNAi transfer (larvae)	Not tested	(70, 71)
Orthoptera	*Teleogryllus oceanicus*	Bacteria°	Injected	Not tested	Enhanced antibacterial activity (male adults)	Reduced son's sperm viability and daughter's ovary mass	(72)

° = alive pathogen;

† = inactivated pathogen. *An extensive description of each of the 57 TGIP articles is available in Supplementary Table 1*.

### The Infection Procedure

The method used to infest the host with the pathogen is chronologically the first step of any experiment. The choice of the procedure of infection and of the pathogen studied orientate and determine the extent to which the analysis and conclusions can be drawn from it. The use of inactivated or living pathogens through either artificial or natural routes of infection should be clearly justified. Considering that these different procedures may generate different outcomes, their relative relevance needs to be taken into account and discussed in regard with the objective of the study; i.e., simply aiming at identifying presence/absence of TGIP or examining its ecological significance under parasitic threat.

#### Injection vs. Ingestion

Most TGIP articles reported parental priming by injection/pricking (61%) while animals were fed with the pathogen in 39% of cases. In only one case, insects (ants *Crematogaster scutellaris*) were dipped in a solution containing the entomopathogenic fungus *Metarhizium anisopliae* to let it attach to the host's external cuticle and naturally infect the host [(59); Supplementary Table 1]. Far from being trifling, the immune response of the host can greatly vary depending on the infection route of the pathogen (73). The choice of the infection procedure must take into account the pathogen biology and ecology, and must be driven by the co-evolutionary interactions between the host and the pathogen (74, 75). One good example comes from *Caenorhabditis elegans* exposed to *Orsay virus*, which is a virus that specifically infects *C. elegans* nematodes by oral route (76). Two articles have been published to study TGIP upon exposure of *C. elegans* to *Orsay virus*: one reported the presence of TGIP after parental larval ingestion of the virus (71) while the other did not present any evidence for improved offspring immunity when *Orsay virus* was injected to adult parents (77).

Many TGIP studies compared different pathogens while using the same infection procedure. They usually drive general conclusions without taking into account the adequacy between the infection procedure and the pathogens studied. This particular point requires specific attention to ensure that no overstated conclusions are driven, which could be misleading and lead to an erroneous view of the universality of TGIP process and mechanisms. Selecting the infection procedures that mimic the natural route of infection should be used wherever possible, as it is the most ecologically relevant and it should maximize the response of the parents and the offspring if TGIP is adaptive. Injection or pricking could be used when the natural route of infection is through mechanical injury, notably to mimic an overload of pathogen associated molecular patterns (PAMPs) in the hemolymph that results from septicemia, but results obtained should be analyzed carefully in regard with the limitations of this technique (see detailed comments in the part below).

#### Inactivated vs. Living Pathogen

Half of TGIP studies used living pathogens for priming the parents while one quarter used specific immunogens, such as peptidoglycans (PGNs) and lipopolysaccharides (LPS), and the other quarter used inactivated pathogens, mostly by heat treatment (Supplementary Table 1). Living pathogens are generally used at a sub-lethal dose to avoid confounding TGIP with the effect of selection (11). This confusion happened in one TGIP study on *D. melanogaster* in which the authors used the LC_50_ (i.e., dose killing half of the population) to prime the parental generation (23). Additionally, one should be careful that the pathogen is not directly transmitted to the offspring, as it could be the case for viruses for example (78, 79). One study concluded that TGIP was observed after exposure of *Plodia interpunctella* larvae to a granulosis DNA virus (29), without ruling out the vertical transmission of the virus that is known to occur in this species (80). In that case, a direct priming of the offspring might be observed in addition to TGIP, which could lead to confounding effects. This phenomenon has not been discussed in TGIP articles yet. Forthcoming studies should first determine if vertical transmission can occur with the pathogen studied, especially for viruses, before willing to investigate any TGIP process.

At the opposite, high concentration of inactivated pathogen is generally injected into the host, which is supposed to mimic an infection in the hemolymph with a massive load of PAMPs. However, it is lacking the response of the host to its pathogenicity. The inactivation procedure itself, notably by heat treatment, can also affect the immunogenicity of the pathogen and the corresponding response of the host, either by increasing the release of PAMPs or by altering their three-dimensional structure (5). Although the presence of PAMPs from uncommon pathogen might trigger some immune response (81), this response might not be complete and might lack all damage-associated immune mechanisms of host response (82–84). Conversely, if TGIP is triggered by a PAMP dose-dependent mechanism, sub-lethal doses of living pathogen might not be sufficient to induce a full within-generation and trans-generational immune priming (5, 85). Results obtained in this case must be very carefully and critically discussed to avoid any over-interpretation that might bias our overall understanding of TGIP in invertebrates. All the limitations associated with this infection procedure (dead pathogen, no damage induced, natural physical barriers bypassed) must be properly acknowledged.

### The Sex of Parents and Offspring

#### The Sex of Parents

Mothers and fathers have been shown to both participate to offspring's immunity; however, this protection can be qualitatively and quantitatively different between the two sexes. This has been evidenced in studies investigating the effect of both mother and father, exposed separately to bacteria or LPS, on the offspring immune status of the lepidopteran *T. ni* (28), the orthopteran *Teleogryllus oceanicus* (72) and the coleopterans *Rhynchophorus ferrugineus* (33), *T. molitor* (34),and *Tribolium castaneum* (41). In 25% of TGIP studies, parents were not separated according to their sex, essentially because they were exposed at the larval stage, at which sex identification can be tricky in invertebrates (Supplementary Table 1). The main problem is that maternal and paternal effects might be confounded. If only one of the two parents is providing most, if not all, trans-generational immune protection, this effect might be diluted and potentially not detected or underestimated.

An additional factor could bias the experiments performed on unseparated sex. In *Drosophila*, males are known to enhance female immunity after mating. This is mediated by the transfer of male seminal fluid proteins (SFPs), activating Imd and Toll pathways, and stimulating antimicrobial peptide (AMP) gene expression in females (86, 87). These SFPs can also affect *Drosophila* female's behavior by decreasing their receptivity to further mating and by increasing egg laying (88, 89), which could also affect the extent of egg immune protection. A role of SFPs on females' immunity and physiology has also been evidenced in several other invertebrate species such as *Aedes aegypti, An. Gambiae*, and *Apis mellifera* (90). This suggests that paternal effects might bias any maternal TGIP if not controlled, and that it should be monitored and quantified (if any) beforehand. To date, the consequence of paternal priming on offspring immune status through mating-associated increased maternal immunity has only been investigated indirectly once. Lytic activity of unchallenged females was similar if they were mated with a challenged or unchallenged male, and offspring's one was not affected by the challenge of any of the two parents (72). Either there is no effect of the father on mother's immunity and of that of their offspring, or the immune parameter measured was not a good reporter parameter for characterization of TGIP in this host-pathogen system. The same procedure followed by McNamara et al. (72), i.e., mating unchallenged females with either challenged or unchallenged males and measuring immune parameters in both the mother and the offspring, should be applied to other species. Other parameters should be monitored, such as expression of immune genes or measurement of prophenoloxidase and antimicrobial activities that are generally more responsive to TGIP. TER parameters should not be omitted, notably offspring survival to challenge with the same pathogen used for paternal priming (8, 34, 56, 65).

Data on TGIP are essentially biased toward the maternal effect (Supplementary Table 1). While parental care theory assumes an important investment of females to the offspring, TGIP derived from fathers may highlight paternal care through cryptic investments (91). Under pathogenic threat, both fathers and mothers may gain benefits from improving their offspring immunity. This protection that offspring receives from mothers and fathers may be more than additive and could result in a general improvement in protection against pathogens. There is a need to increase the number of studies including paternal effect to provide a more comprehensive view of the sex-dependent TGIP process (41, 42, 47).

#### The Sex of Offspring

In the oceanic field cricket, *T. oceanicus*, the antibacterial immune response of male offspring was mediated by a complex interaction between maternal and paternal immune status (72). Moreover, a sexually dimorphic TGIP was found, as female offspring did not exhibit immune protection when male offspring did (72). Sex-specific changes in the expression of some immune-related genes have been observed in offspring *Manduca sexta* larvae from parents challenged with *E. coli* and *Serratia entomophila*. In the same study, they also observed a significant increase in histone acetylation in male offspring larvae but not in females upon parental exposure to *S. entomophila* (64). Significant differences in gene methylation between offspring sexes was also observed (64). These observations suggest that both parental and offspring sex can induce contrasting TGIP phenotypes. Males and females differing in their susceptibility to infection is very common if not universal (92, 93). Therefore, TGIP measures without controlling the sex of the offspring (and of the parents) can be very complex and even lead to misinterpretation of the phenotype observed. This has been largely neglected in TGIP so far and should receive a much greater interest in the future.

### The Developmental Stage

The life cycle of invertebrates is constituted of a sequence of several developmental stages that strongly differ in terms of metabolism, physiology, and immunity. Therefore, the choice of the developmental stage of the parents for priming and of the offspring for measuring the outcome of TGIP is far from being trivial. The choice of a specific developmental stage for the priming of the parents has often been driven by the adequacy to the pathogens used and by the easiness of their manipulation (Supplementary Table 1). For the choice of the offspring developmental stage, most articles focused on a unique specific stage, which can have consequences on the phenotype observed and on the conclusion of the study. Generally, offspring were studied at the same stage at which parents were exposed, which is the most ecologically relevant, or in the egg to study the effect of TGIP at the very first steps of offspring development (36, 40, 56, 63).

In the mollusk *Chlamys farreri*, the immunity of the offspring from mothers exposed to the bacterial pathogen *Vibrio anguillarum* was studied at different ontogenic stages (4-cell, blastula, gastrula, trochophore) from egg to larva (67). It showed that antibacterial activities, the expression of genes encoding immune effectors and an enzyme of the antioxidant system, the superoxide dismutase (SOD), differed depending on the stage at which they were measured (67). In the moth *M. sexta*, monitoring of the melanisation index, lysozyme and antimicrobial activities in offspring from PGN-primed parents revealed that there was a high fluctuation (from 2-to 100-fold) of these parameters between different larval instars, the pupal and the adult stages (61). Focusing only on a limited number of offspring developmental stages increases the risk of missing the main TGIP effect. Another example comes from the Gastropoda *Biomphalaria glabrata* in which no TGIP has been found after parental exposure to the metazoan parasite *Schistosoma mansoni* in 10-day and 60-day old offspring (B. Gourbal, unpublished data). TGIP should be a selected mechanism in this species considering that it exhibits a low dispersion and can live up to several months (19) and that evidence for maternal protection by transfer of immune proteins from naïve females to their eggs has already been reported (15, 94). One could then argue that studies reporting absence of TGIP might just have missed the developmental stage at which it is expressed. This highlights the importance of following offspring's immunity at different life stages, from egg to adult, and selecting the good proxy for identifying TGIP.

Another important factor to take into account is the time elapsed since the parental priming was performed, as the effect of priming in the mother might not be stable over time and may influence the immune transfer to the offspring. Alternatively, these changes might just be byproducts of the fluctuating immunity of the mother passively transferring effectors to her eggs. Such a phenomenon has been characterized in *T. molitor*, in which the antibacterial activity in mother's hemolymph decreased each day since the priming occurred until it was back to ground level at the tenth day (36). They observed that the transfer of this antibacterial activity to the eggs was 1-day delayed and that eggs older than 9 days exhibited a significantly decreased antibacterial activity until none was detected after 11 days (36). Therefore, TGIP must be considered as a dynamic process with a temporal dimension that experiments focusing on a unique life stage might miss.

## Ecology and Evolution of TGIP

TGIP exhibits variable characteristics according to host and parasite/pathogen species, which raises numerous questions related to its evolutionary ecology. In particular, its adaptive nature conditioning its evolution is debated. Moreover, the epidemiological consequences of TGIP and its impact on the evolution of parasite/pathogen virulence have only started to be studied.

### Is TGIP Adaptive?

An important issue in the study of the ecology and evolution of TGIP is to know whether it is adaptive. The criteria required to characterize adaptive parental effects, often called maternal effects, can be used to describe the adaptive nature of TGIP (95). *A priori*, TGIP would be adaptive if it is a response to an enhanced risk of infection in the parental environment by a virulent pathogen that is likely to persist in the offspring environment. However, exposure to the virulent pathogen should be relatively rare to prevent the evolution of enhanced basal resistance to infection (96, 97). Hence, TGIP is expected to be adaptive when mothers sense environmental cues that predict higher risk of attacks by a virulent pathogen. This would reduce offspring fitness, inducing appropriate phenotypic changes in offspring that increase their fitness in this new environmental condition (98). This implies that phenotypic changes and associated offspring performance in the offspring should be specific to the new environmental condition (99). Therefore, TGIP expression should bear costs with strong negative implications for offspring fitness in mismatched environments. Finally, as adaptive TGIP is expected to be shaped by natural selection, it should be genetically based and show genetic variation in its expression. So far, the environmental predictability of pathogen attacks between host generations received little consideration. While there is evidence that TGIP can benefit the offspring and bear costs in artificial conditions, implication for host fitness in ecologically relevant contexts is limited. Evidence for specific TGIP often suffers from inappropriate experimental design, and the potential genetic bases behind TGIP expression have never been investigated. These different aspects are discussed below.

#### Detection of Reliable Cues Predicting the Risk of Pathogen Attacks Between Generations

As a form of adaptive trans-generational plasticity, TGIP is expected to evolve from changes in the risk of pathogen attacks between generations that reduce the fitness of parents by reducing that of the offspring. There is compelling evidence that exposure to pathogens decreases the fitness of their invertebrate hosts (100). An increased risk of infection in the offspring generation compared to the parental one is therefore expected to negatively affect offspring fitness.

Parents should be able to sense cues that predict a higher risk of attacks by a pathogen persisting in the offspring environment. There is evidence that Pathogen/Danger Associated Molecular Patterns (PAMPS and DAMPs) of microbes are perceived by the invertebrate immune system (101). Pathogen attacks might be a reliable cue reflecting an enhanced probability of future infection as it might indicate that the pathogen is becoming more abundant and could persist in the environment (102). However, this may depend on the pathogen. Parents must be able to properly sense these cues and appropriately adjust the phenotype of their offspring to match the new environmental condition. As seen above, several studies have reported improved survival of the offspring of some invertebrates when the parental generation has been exposed to a multitude of immunogenic stimulations before reproduction (5, 103). However, whether these maternal effects on offspring resistance are indeed adaptive or merely physiological inevitabilities is still unclear. Indeed, most studies manipulate the parental immune status without explicitly clarifying whether that manipulation represents a reliable signal that parents can sense to predict the environmental state of offspring. It is important that variability and predictability of the pathogenic environment across host generations is relevant of the ecology of the study organisms (99). Therefore, pathogens that do not belong to the range of pathogens naturally occurring in the host environment or that are not able to persist long enough in the offspring environment are unlikely to stimulate TGIP if it is adaptive (37, 39).

#### Costs of TGIP

Whilst TGIP appears beneficial when the parental condition persists over the next generation, its inducible aspect—the fact that the enhanced offspring immunity is induced by the parental exposure to the pathogen—suggests it is also costly. Indeed, in the absence of cost, selection would likely favor elevated basal levels of immune defense in the offspring and there would be no priming response. Immunity is known to trade-off against other costly life history traits (101). Hence, fitness costs of TGIP may exist and outweigh its benefit when the parental condition are unlikely to persist in the offspring environment (75). Moreover, the fact that TGIP can occur despite bearing fitness costs would be another strong evidence in favor of the adaptive nature of TGIP.

There is evidence of potential fitness costs associated with TGIP for mothers. For instance, bacterially immune-challenged females of *T. molitor* transiently lay a variable number of eggs with internal antibacterial activity that is traded-off against the total number of eggs produced (36). Furthermore, the amount of antibacterial activity in the eggs also negatively correlates with that in mothers' hemolymph (35). Hence, the inducible transfer of antibacterial activity to the eggs appears to bear significant costs for the mothers, otherwise they would be able to protect all the eggs of all their clutches without impairing their own immunity. Immune-challenged females should therefore adjust and optimize their investment into TGIP, their fecundity and their immunity; they should balance between their perception of their own risk of dying from the infection and the expected persistence of the parasite to maximize offspring fitness (36).

TGIP is expected to be costly for the offspring too as they may trade-off enhanced immunity with other important life history traits, such as growth in the beetles *T. castaneum* (41) and *T. molitor* (36, 39), and reproduction in the moth *M. sexta* (61). Costs of TGIP may also involve trade-offs between arms of the offspring immune system. This is likely the case of the daughter workers of bacterially immune-challenged queens of the bumblebee, *B. terrestris*, exhibiting enhanced immunity against a bacterial infection but reduced resistance to the trypanosome parasite, *Crithidia bombi* (57). The costs associated with TGIP in the offspring are likely to have strong negative implications for offspring fitness if the parental conditions do not persist in the offspring environment. As mentioned above, these negative effects due to TGIP in the offspring are often assumed to result from the offspring trading-off their immunity against other important functions. However, it is difficult to state whether these costs arise from such a trade-off and/or from a reduced parental investment per offspring resulting from the cost of the parental immune challenge. In the latter case, reduced parental investment into their progeny should be observed early in the offspring life. However, recent evidence in *M. sexta* (63) and *T. molitor* (40) showed that immune challenged females produced eggs with the highest hatching success. Furthermore, in *T. molitor*, the resulting young larvae show enhanced survival to starvation within the first month post hatching (40), although they are known to exhibit prolonged developmental time (34, 40). Therefore, this suggests that the latter cost paid by the offspring likely arises from TGIP and not from a reduced parental investment to the offspring. Now, whether these costs associated with the expression of TGIP significantly affects the host fitness in a mismatched environment has never been tested so far.

Assuming that TGIP is an important mean of defense against repeated infections by certain pathogens and that the above costs associated to the expression of TGIP could significantly affect host fitness, a reduction of these costs is expected to evolve in parallel to the evolution of TGIP. Under this hypothesis, TGIP response would reveal less costly in response to the most threatening pathogens. TGIP responses in maternally-primed offspring of *T. molitor* with Gram-positive bacteria resulted in higher protection and lower prolonged developmental time than in maternally-primed offspring with Gram-negative bacteria, suggesting that Gram-positive bacteria might have been a strong selective force behind the evolution of TGIP in this insect species (39).

#### Specific Phenotypic Adjustment in Offspring to Face the Expected Parasitic Conditions

**Figure 3:**
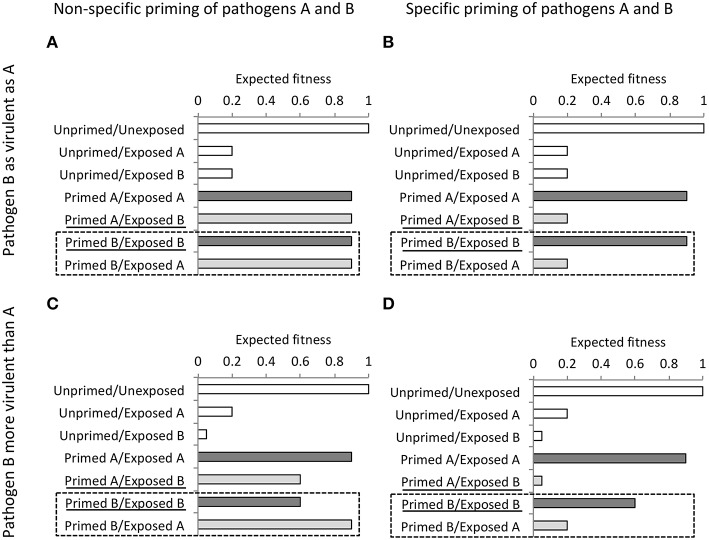
Expected fitness outcomes (arbitrary values) of maternally unprimed and primed offspring with a pathogen A or B upon exposure to pathogen A or B when maternal exposure to pathogen A and B induces non-specific (left panels) or specific (right panels) TGIP effects in the offspring and when pathogen A and B exhibit similar (upper panels) or different virulence (lower panels). Experiments testing the specificity of TGIP effects without reciprocal combinations of maternal and offspring exposure to pathogens A and B may lead to a wrong conclusions as explained below. A first case is when an experiment uses maternally primed offspring by one pathogen only (here pathogen A), and tests offspring fitness when exposing to the same pathogen and at least another one (here pathogens A and B). Such an experiment, therefore, omits the 2 combinations of Priming/Exposure boxed with a dashed line (here: Primed B/Exposed B and Primed B/Exposed A). In that case, only results from the situation illustrated in **(A)**, when pathogens A and B induce non-specific priming and when these pathogens exhibit similar virulence, can be conclusive. Indeed, without the results from the reciprocal combinations illustrated in the box with a dashed line, it is uncertain whether pathogen B induces priming in **(B)**, and it is not possible to tell whether the results in **(C,D)** may result from specific TGIP of difference in virulence between pathogen A and B. Another case is when an experiment uses maternally primed offspring by several pathogens (here pathogens A and B), and tests their fitness when they are exposed to only one of the pathogens used for maternal priming (here pathogen A). Such an experiment then misses results from the underlined “Priming/Exposure” combinations (here Primed A/Exposed B and Primed B/Exposed B). This approach is insufficient too to examine specificity of TGIP as it only allows being conclusive on the unspecific TGIP effects of pathogen B whereas specificity of TGIP by pathogen A remains unknown in situations illustrated in **(A,C)**, and it is uncertain whether pathogen B induces priming in **(B,C)**.

If TGIP constitutes a form of adaptive phenotypic plasticity, a central prediction drawn from adaptive maternal effects (104) is that offspring from mothers anticipating an enhanced risk of attack by pathogen A will perform better when exposed to the same pathogen than to another pathogen B. This suggests that TGIP exhibits a certain level of specificity to pathogens. This also suggests that solely testing the performance of the primed offspring compared to controls is not sufficient to address the question of the adaptive significance of TGIP. Thus, appropriately testing whether TGIP has evolved as a mean of adaptive trans-generational plasticity requires reciprocal full factorial experiments. These experiments test the performance of offspring, originating from mothers challenged with either pathogen A and B, hence are all primed with either pathogen A or B, in addition to controls. Figure 3 illustrates what outcome from a reciprocal full factorial experiment are expected if TGIP is specific, or not specific to pathogens that exhibit either similar or different virulence. In the light of this theoretical model, we can reconsider and adjust the conclusions drawn from two published partial approaches that aimed at investigating the specificity of TGIP (32, 49). On the one hand, partial experimental approaches in which the performance of maternally-primed offspring with a pathogen A is tested by exposing them to pathogens A and B cannot be conclusive on the specificity of TGIP. In that case, the results that would suggest a specificity can also be explained by confounding factors, such as the potential inability of pathogen B to induce priming (Figure 3B) or the potential difference in virulence induced by the pathogens A and B (Figures 3C,D). Therefore, the use of such a partial approach cannot be conclusive on the pathogen-specific effects of TGIP (49). On the other hand, opposite partial approaches, in which the performance of maternally-primed offspring are exposed to only one of the pathogens used for maternal priming, is also limited to test whether TGIP is specific (Figure 3). It can, at best, be conclusive on the potential non-specific TGIP effect induced by the pathogens used for maternal priming only when they have similar virulence (Figure 3A). In the other situations, variation of virulence between pathogens and the potential inability of at least one of the pathogens to induce TGIP can explain these results too. This possibility cannot be excluded without conducting the missing reciprocal combinations of maternal priming and offspring challenge [Figure 3; (32)]. The use of a fully reciprocal factorial experimental design of maternal priming and offspring exposure to *E. coli* and *B. thuringiensis* successfully evidenced a level of specificity in the expression of TGIP in the red flour beetle, *T. castaneum* (41). This latter study indeed showed that primed offspring exposed to the same bacterial pathogen as their parents exhibited lower mortality than when they are exposed to the other bacterial pathogen. It also showed that the expression of TGIP, in terms of offspring resistance to infection, was more variable in *E. coli*-primed offspring than in individuals primed with the natural pathogen *B. thuringiensis* (41). Hence, in addition to the use of a fully reciprocal factorial experimental design of maternal priming and offspring exposure to pathogens, the use of procedures of host exposure to pathogens relevant of those naturally occurring appears essential to provide a comprehensive understanding of the adaptive nature of TGIP and of the selective forces at the origin of its evolution.

#### Genetic Bases of TGIP

If adaptive, TGIP should have been shaped by the action of natural selection and its expression should be genetically encoded and therefore heritable. Ample additive genetic variance and heritability were found for components of immunity and life history traits in insects (105–107). Genetic variance for TGIP might therefore be expected as well. Host populations likely face substantial spatial and temporal variation of the pathogen diversity, pathogen abundance, and resource availability that altogether modulate the strength of selection on TGIP. Thus, TGIP is expected to show variation in its expression as it also imposes fitness costs, which generate trade-offs with life-history traits (34–36, 39, 41). In line with this, evidence for substantial variation in TGIP responses among natural populations of *T. castaneum* to its natural pathogen, *B. thuringiensis*, was found (45). Furthermore, significant inter-individual variation in their investment into the immune protection of their eggs in relation to their fecundity were identified in immune-challenged females of *T. molitor*, suggesting different strategies of investment into TGIP (36). It is yet unknown whether such a variation in investment into TGIP and its covariation with other fitness-related traits have genetic bases. Therefore, measuring its heritability and estimating potential genetic correlations with other life-history traits appears of primary importance. It would allow for inferring about how much natural selection could act on this aspect of invertebrate immunity to reliably understand its evolution. So far, this has never been investigated.

### Evolution of TGIP

While TGIP may confer a large fitness advantage, it does not seem to be universal as studies have failed to detect it in some invertebrate groups (22, 23, 25). Others have even found a negative effect of the maternal challenge on the offspring resistance to infection (24). However, the limited number of taxa investigated might bias this view (Figure 2). Assuming that all organisms have the potential to evolve immune priming, its associated fitness costs may prevent its selection, depending on the biology and ecology of the species. It is also remarkable that in invertebrate species for which TGIP exists, a restricted range of parasites or pathogens induce its expression (Supplementary Table 1). Furthermore, depending on the parasites/pathogens involved, the TGIP response can be either non-specific, leading to cross-immunity, or specific toward the pathogen that challenged the parental host (41, 50). Hence, the expression and specificity of TGIP seem to depend on the host-parasite system involved.

Because TGIP is expected to provide protection against repeated infections, its evolution should depend on the risk of subsequent infections in the offspring generation after a parental contact with a given parasite/pathogen. Therefore, it is expected to be a selected process in species with a relatively long life-span and limited dispersion, increasing the chances of the offspring encountering a pathogen diversity similar to the one experienced by their parents (19). While such a risk might be conditioned by host-life history characteristics, it may also depend on parasite/pathogen traits that determine the severity of disease. On the one hand, avirulent parasites/pathogens should not select for TGIP. On the other hand, highly virulent ones are not expected to promote the evolution of TGIP if they induce host death before they have the opportunity to reproduce (19). However, this may depend on whether TGIP could be initiated by non-infectious parental contact with the parasite/pathogen, that is when the parents are exposed to a low dose of the disease agent without becoming infected (29, 108). Hence, depending on the priming mechanisms of susceptible parents (through either infectious or non-infectious pathogen encounters), intermediate-to-high virulence levels of parasite/pathogen are expected to favor the evolution of TGIP (46).

### Consequences of TGIP on Pathogen Virulence Evolution

While parasite/pathogen virulence is likely an important factor for the evolution of immune priming within and across generations (19, 109, 110), the influence of immune priming on the evolution of virulence has rarely been evoked. If immune priming does not confer full immunity but rather prevents hosts from dying quickly from the infection, it contributes in extending the period during which parasites/pathogens may replicate and be transmitted. Studies from adaptive immunity of vertebrates suggest that imperfect vaccines may promote the evolution of more virulent pathogens (111, 112). As such, a high rate of primed individuals has the potential to maintain a high number of virulent pathogens in a host population that can spread to susceptible populations. Further investigation of the underlying mechanisms of immune priming and its impact on host survival to disease is needed to better understand their role in the evolution of pathogen virulence (75).

## The Many Roads to TGIP: Hypothetical Scenarios Based on Empirical Data

Many molecular actors of the innate and acquired immunity of invertebrates have been identified. Consequently, many TGIP studies monitored several of these known mechanisms by measuring their activity, such as lysozyme, antimicrobial or phenoloxidase (PO) activities, or their gene expression by RT-qPCR (reverse transcription quantitative PCR) (Table 1; Supplementary Table 1). Only few studies used global approaches to unravel the potential role of other genes and proteins, by using next-generation sequencing (NGS) transcriptomic approach by RNA-seq (46, 58, 69), or by proteomic profiling using 1-dimension (28, 37) or 2D polyacrylamide gel electrophoresis (SDS-PAGE) coupled with mass spectrometry (MS) analysis (60). In future studies, such global approaches should be more widely adopted to identify additional candidates that could be specific to TGIP and have not yet been identified in within-generation immune priming. It would also be helpful to identify potential metabolic reorganization following an increased immunity, notably due to energy reallocation processes.

**Figure 4:**
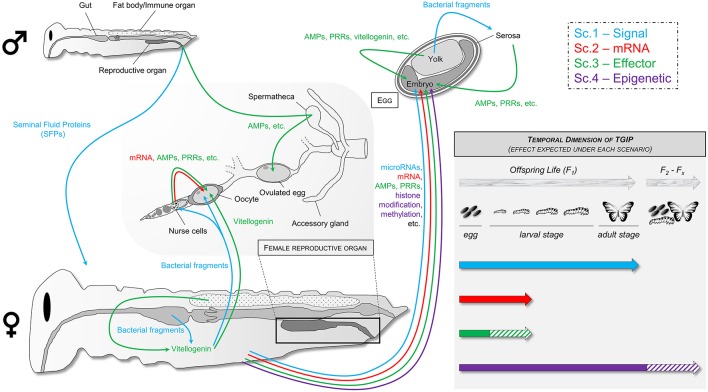
Hypothetical mechanisms responsible for TGIP in invertebrates following the four described scenarios highlighted in blue, red, green, and purple for scenarios 1, 2, 3, and 4, respectively.

In light of the different potential mechanisms identified, we propose four different hypothetical scenarios to explain how TGIP can occur and how the underlying mechanisms can be characterized (Figure 4). Although there might be as many mechanisms as there are invertebrate/pathogen combinations, the objective of such scenarios is to highlight common features and to provide a baseline to facilitate further discussions about TGIP mechanisms and processes. These scenarios are by essence not mutually exclusive and could act simultaneously at the same developmental stage of the offspring and/or act sequentially at different stage of the offspring life (Figure 4).

### Scenario 1—Transfer of Signal(s)

In the first scenario, parents may transmit a “signal” to their progeny, which could be an eliciting substance transferred in the developing eggs. Such a signal can notably be bacterial peptides translocated from mother's gut to the egg. This phenomenon has been characterized and visualized by fluorescence microscopy in *M. sexta, G. mellonella*, and *T. castaneum* mothers exposed to bacteria and it was associated with an increased expression of immune genes in the eggs (43, 60, 64). In *A. mellifera*, it has been hypothesized that such translocation was mediated by vitellogenin (113, 114). Vitellogenin would recognize bacteria by specifically binding to pathogen-associated molecular patterns (PAMPs), such as PGN and LPS, to trigger the transfer of cell-wall fragments of bacteria into the eggs (113). It could also specifically recognize and transfer bacterial fragments from the gut up to the workers glands producing the royal jelly and eventually to the eggs produced by the queen (114), which could participate to the social immunization in this species (9). In *G. mellonella* and *T. castaneum*, although such transfer was not associated with vitellogenin at this time, the route followed by the bacterial proteins had been identified (i.e., crossing the midgut epithelium then being entrapped into nodules in the hemocoel followed by an accumulation in the ovaries ended by a deposition in the eggs) and could match with the tropism and mechanism of translocation of vitellogenin into the eggs (60, 115). Such mechanism would expose the developing embryo within the egg to PAMPs from pathogens that his mother encountered during her life. It would be an easy way to induce an immune priming in the offspring in order to boost its innate immunity and increase its capacity to respond to the pathogen community to which it might be exposed after hatching. It is, however, yet to be characterized whether this mechanism is strictly passive or whether the mother can actively stimulate vitellogenin production and/or activity in response to pathogen exposure, and whether it can be facilitated by a potential paternal effect. Nevertheless, one should be cautious with this hypothesis considering that similar experiments performed using *M. sexta* exposed to *S. marescens* failed to identify such bacterial transfer (63). Intriguingly, bacterial translocation has been recently identified in the same insect species to a bacteria from the same genus, *S. entomophila*, but this discrepancy with the previous study was not acknowledged nor discussed (64). Therefore, additional evidences have to be accumulated from different teams using complementary approaches to decipher the exact mechanisms using similar host-pathogen combinations. Similar experiments should also be performed with different insect-pathogens couples, including viruses, fungi, microsporidia, and protozoan parasites, to know whether translocation of pathogen proteins is a generalist mechanism or a bacteria-specific TGIP mechanism and determine its occurrence in the tree of life.

Transfer of maternal microRNAs that directly act on offspring gene expression could also be a “signal” triggering TGIP (116). The involvement of microRNAs has not been investigated in TGIP yet, despite their known role in invertebrate immunity and host-pathogens interaction (117, 118). Such signals could induce the activation of immune-related genes in the developing embryo inside the egg and/or by the extraembryonic serosa, which is a frontier epithelium able to express many immune genes and provide the insect egg with a full-range innate immune response (119, 120).

### Scenario 2—Transfer of mRNA(s)

In the second scenario, females may provide their eggs with mRNAs coding for key antimicrobial immune effectors, which are then produced by the developing embryo and/or by the serosa surrounding the embryo (120). Transfer of maternal mRNAs in developing eggs during oogenesis has been characterized in many species, including insects (121). In insects with polytrophic meroistic (e.g., Hymenoptera, Lepidoptera, and Diptera) and telotrophic ovaries (e.g., Hemiptera and Coleoptera), these maternal mRNAs are synthetized by nurse cells and are provided to oocytes via the trophic cord (122–124). Although these mRNAs are mostly known to be involved in the control of development (125), they may also serve for early immune protection of embryos. In the fish *Cyprinus carpio* L. for example, maternal mRNAs encoding immune-related genes have been identified in unfertilized eggs (126).

### Scenario 3—Transfer of Effector(s)

In the third scenario, females could directly transfer immune effector proteins to their eggs, either passively through the diffusion or sequestration into the egg of proteins present in the mother's hemolymph (scenario 3a), or actively via the provision of eggs by specialized cells, such as nurse cells that are known to produce proteins transferred to oocytes (121) (scenario 3b). Transfer of immune effectors is well-known in vertebrates where antibodies are transmitted through the yolk in birds, fishes, and reptiles, or through the placenta or milk in mammals (3). Although antibodies do not exist in invertebrates, other immune effectors can be transferred to the offspring such as lectins, LBP/BPI (LPS-binding proteins/bactericidal permeability-increasing proteins) and antimicrobial peptides (AMPs) (15, 94).

#### Antimicrobial Peptides

The involvement of AMPs in TGIP has been extensively investigated, as they are central in invertebrate immunity. A wide range of AMPs that act against a large number of pathogens can be produced and the majority of AMPs have been found in more than two invertebrate orders (127, 128). Several studies reported the transfer and storage of AMPs from mothers into the eggs (129–131). Mother-derived AMPs have notably been shown to condition the colonization of the embryo by symbiotic bacteria (132). The involvement of AMPs in TGIP has only been investigated in Coleoptera, Lepidoptera, and Hymenoptera after parental exposure to LPS, PGN, bacteria or fungi (Supplementary Table 1). Increased AMP gene expression is not triggered by all pathogen challenges and the set of AMPs differentially regulated differs from one pathogen to another in offspring from challenged mother compared to offspring from unchallenged mother (28, 42, 43, 60, 62, 64, 68).

Despite such variability in the results obtained, several key candidate AMPs have already successfully been identified. Among them, gloverin is a serious candidate that was found over-expressed in eggs from mothers primed with PGN and larvae from mothers exposed with *E. coli* in *M. sexta* (62, 64), in *Galleria mellonella* eggs from mothers exposed to *S. entomophila* (60), and in *T. ni* larval offspring from mothers fed with a mixture of *E. coli* and *M. luteus* (28). Gloverin is a lepidopteran-specific AMP that has been implicated in antibacterial and antifungal response in several lepidopteran species (128) and its involvement in TGIP clearly deserves further in-depth investigation. In *T. molitor*, a defensin-like AMP (tenecin-1) was systematically found in egg extracts from mothers injected with different bacteria (*A. globiformis, B. thuringiensis, E. coli*, and *S. entomophila*) but was absent in eggs from unchallenged mothers (37). Next-generation RNA sequencing (RNA-seq), which gives access to the entire transcriptome of species, allowed for extending the list of candidate AMPs that will require further investigation (46, 58). Dedicated studies focusing on AMPs through pluridisciplinary approaches aiming at characterizing their involvement up to the functional level are now needed.

#### Vitellogenin, a Multi-tool Protein

Apart from its potential role as a bacterial peptide translocator (see scenario 1), vitellogenin can play many additional roles. Vitellogenin is a highly evolutionarily conserved protein whose main role is to provide the embryo with sufficient energetic resources for its proper development within the egg (133). Vitellogenin can also play an important direct and indirect role in the defense of invertebrates against stress and infections. In response to an oxidative stress, honeybees (*A. mellifera*) are synthesizing a high quantity of vitellogenin that is able to recognize damaged cells and bind to living cells to protect them from reactive oxygen species (134, 135). Vitellogenin has also been implicated in the modulation of the immune response of invertebrates, notably indirectly due to shared gene expression regulation regions with AMPs, such as defensins (136, 137). Vitellogenin can also directly act as a multivalent pattern recognition receptor (PRR) with an opsonic and antibacterial activity (138, 139). Due to its potential involvement at different steps of the anti-pathogen response and in the translocation of pathogens' PAMPs into the eggs, the role of vitellogenin in TGIP requires extensive investigation, notably by different teams on different biological systems. This would allow for verifying if its involvement is specific to a restricted list of host/pathogen combinations or if it is a general mechanism, at least in oviparous species. However, studying the involvement of vitellogenin in TGIP will be a complex task, notably due to its many roles in the physiology, metabolism and/or immunity of invertebrates and will require a specific investigation to properly address and disentangle the many confounding effects.

### Scenario 4—Epigenetic Modification(s)

In the fourth and last scenario, parents exposed to a pathogen would experience an epigenetic reprogramming (e.g., by acetylation/deacetylation of histone and/or by methylation/demethylation of immune genes) and would transfer this reshaped epigenetic state to their offspring. Modification of gene methylation and histone acetylation are major epigenetic factors that can boost or impair invertebrate immune response toward bacteria, viruses, or fungi (140, 141). Surprisingly, only a limited number of studies investigated the role of epigenetics in TGIP. One focused on histone acetylation in the response of the crustacean *Artemia sp*. to *Vibrio campbelli* (49) and two others on gene methylation after bacterial exposure of *T. castaneum* (43) and of *T. molitor* (38). While TGIP was identified in both cases, no link between epigenetic modifications and TGIP was found. More recent studies tend, however, to point out some role of epigenetic in TGIP. In *T. castaneum*, priming of fathers with *B. thuringiensis* combined with RNAi of a DNA methyltransferase (*Dnmt2*), known to drive CpG methylation on tRNA (142), led to a low (~10%) although significant decrease in offspring survival to the same pathogen (143). However, the mechanism by which tRNA methylation could participate to TGIP is still unclear and further investigation is now required. In a recent comprehensive study, Gegner et al. (64) reported evidence for a sex-specific modification in the DNA methylation and histone acetylation in *M. sexta* offspring larvae from parents exposed to pathogenic *S. entomophila* and non-pathogenic *E. coli*. The authors argue that such modifications are associated with the differential expression of immune-related genes that they measured in offspring, without however providing evidence for a clear linkage between epigenetic modifications and altered immune gene expression (64). Based on these contrasting results on different host-pathogen combinations, it is impossible to draw an overall picture of the implication of epigenetic in TGIP yet. Considering the prominent role of epigenetics in many trans-generational adaptation processes in animals and its implication in the modulation of several within-generation immune response pathways, its involvement in TGIP must be more deeply and widely investigated (116, 144, 145). It could be a notably good candidate to explain at least a part of the paternal effect and of the sustenance of TGIP effect over multiple successive generations (47, 48).

## How to Experimentally Disentangle the Different Scenarios?

In this part, we describe how the experiments should be designed and their outcome interpreted to decipher between the different scenarios of TGIP. Although it is impossible to be exhaustive and to provide guidelines universal for all host-pathogen combinations, our aim is to present some key parameters to be particularly monitored. Noteworthy, considering that more than one mechanism belonging to at least two scenarios could act simultaneously, we focus on those parameters that allow proceeding by elimination to get to the scenarios most likely involved in TGIP (Table 2).

**Table 2:** Expected presence of transcripts and proteins in immune-challenged females and their eggs according to the four different scenarios.

	**Transfer of a signa**l **(scenario 1)**	**Transfer of mRNA** **(scenario 2)**	**Transfer of effectors** **(scenario 3)**		**Epigenetic shaping** **(scenario 4)**
			**a. Passive diffusion**	**b. Active transfer**	
Gene expression in mothers	Not necessarily	Yes, in ovaries	Yes, in fat body and/or hemocytes	Yes, in ovaries	Not necessarily
Presence of the protein in mothers	Not necessarily	Not necessarily	Yes, in the hemolymph	Yes, in ovaries	Not necessarily
Transcripts in embryo	Yes, in nuclei of embryo cells and/or in serosa	Yes (maternal origin) but not necessarily in nuclei of embryo cells	No	No	Not necessarily
Presence of the protein in eggs	Yes	Yes	Yes	Yes	Not necessarily

Under these four different scenarios, transcripts coding for the immune effector(s) found in the eggs and the effectors themselves are expected to localize in distinct parts of the mother's and egg's tissues (Table 2). In the case of a maternal transfer of immune effectors (scenario 3), quantity of transcripts of the effector(s) should not be elevated in the eggs laid by immune-challenged females, while under scenarios 1 and 2, transcripts should be detected at an abnormally high level in the eggs from primed mothers compared to unprimed ones (Table 2). However, in the case of a maternal transfer of mRNAs, quantity of transcripts should not be elevated in the oocyte nucleus (i.e., the site of transcription in oocytes), but it should be increased in maternal tissues, such as the nurse cells and trophic cords, and/or in more systemic mother's organs.

Besides, absence of transferred transcripts in females would favor the hypothesis of a transfer of a maternal signal (scenario 1), while their presence may not help to distinguish between the scenarios 2 and 3 (Table 2). The precise localization of these transcripts may however be informative. In insects, many antimicrobial effectors are known to be expressed in the fat body and in hemocytes following an immune challenge (146, 147), but one should be aware that some can be expressed in other tissues. For example, the AMP drosocin is expressed in the calyx and oviducts of mated *D. melanogaster* females that have started to lay eggs (148) and in the medfly, *Ceratitis capitata*, ceratotoxin A and B are expressed constitutively within the female's accessory glands (149). In the case of a transfer of maternal mRNAs (scenario 2) or of an active transfer of effectors (scenario 3b), high levels of transcripts are expected to be observed in the ovaries, especially in the nurse cells known to provide both maternal mRNAs and proteins to developing oocytes, and in the trophic cords, which connect the nurse cells to the oocytes.

The presence of large amounts of effector proteins in female tissues would rather favor the third scenario while their absence would clearly favor the scenarios 1 and 2 (Table 2). Under the third scenario, the presence of these molecules in the mother's hemolymph would favor the hypothesis of a passive transfer of proteins (scenario 3a) while a higher concentration within ovarian tissues would rather indicate an active transfer (scenario 3b).

The outcome of the fourth scenario, involving epigenetic reshaping, is difficult to predict in term of transcript and protein presence in mother and offspring as it would largely depend on the gene(s)/protein(s) that are affected (Table 2). It would require a specific investigation through dedicated approaches (e.g., chromatine immunoprecipitation sequencing (ChIP-seq) and bisulfite sequencing (BS-seq) for studying DNA-chromatine interaction and methylation, respectively). Nevertheless, if the immune protection is maintained across several successive generations, this would strongly indicate that an epigenetic factor is involved (Figure 4). However, it would not completely exclude the other scenarios, as the increased immune status of the offspring due to an increased amount of proteins could be transferrable to the next generation(s), which would support the involvement of the transfer of effectors too (scenario 3).

Host-associated microbiota can affect the fitness of its host in a number of ways, including the modification of host-parasite interactions, and thus, the outcome of disease. Intriguingly, the role of microbiota in TGIP has never been investigated despite its pivotal role in immune priming (14). There are increasing evidences that microbiota can affect the host response to pathogens by directly competing with them and/or by modulating the host innate immunity (14, 150). In reciprocity, results have accumulated to support the effect of the host immune system on microbiota homeostasis. Therefore, different individuals of the same species with the same genetic background but with different microbiota could mount different immune responses against a pathogen upon infection, which could, in turn, differentially shape their microbiota. This might affect their ability to transfer this immunity to their offspring; notably if the microbiota is transgenerational acquired, a change in parents' microbiota could affect offspring's one. Moreover, any of the mechanisms of parental immune transfer cited in the above four scenarios could directly modulate the offspring innate immunity and/or have indirect effect by modifying offspring microbiota. For example, AMPs are known to shape offspring microbiota in many species. An increase of their quantity in offspring will increase its immune response capacity and meanwhile alter its microbiota, which could also enhance or mitigate the direct TGIP effect. This would render the outcome of TGIP hardly predictable and quantifiable. Considering that microbiota was shown to be mandatory for immune priming in some species (151, 152), specifically investigating its role in TGIP is of utmost significance.

## Guidelines for Further Studying TGIP in Invertebrates

1. Trans-generational immune priming corresponds to the plastic adjustment of offspring immunity, as a result of parental immune experience. It represents a recent field of research (20 years old) and it has been increasingly studied during the last years. Investigating new invertebrate species is required to provide key information on the occurrence of TGIP in the tree of life.
2. Before conducting any experiment on TGIP (or on any other topic), one should consider whether the experimental design and, more importantly, the statistical analysis pipeline are adapted to address the question raised. Too many TGIP articles suffer from a flawed experimental design and/or a non-adapted statistical analysis. For investigating the specificity of TGIP, the use of a fully reciprocal factorial experimental design is mandatory.
3. Considering that TGIP can be costly for fitness of both parents and offspring, it is expected to occur principally against the most threatening and recurrent pathogens from their environment. Therefore, characterizing the ecology of the host before studying TGIP is an important prerequisite to select the most appropriate pathogen(s) for studying TGIP and to avoid missing the phenotype due to an inadequate host-pathogen combination. Investigating TGIP in host-pathogen combination that is not expected to trigger TGIP would also be required to test the assumptions about its presence.
4. The infection procedure (ingestion/injection and inactivated/living pathogen) and the dose applied must be chosen based on their adequacy to the biology and ecology of both the host and the pathogen studied, which must be characterized beforehand. When comparing two pathogens, these pathogens must share some common features in terms of infection route and pathogenicity to be comparable through the same infection procedure.
5. When possible, immune parameters and associated fitness costs should be measured separately in females and males in both parents and offspring to disentangle sex biased TGIP. Ideally, the paternal influence should be investigated more in-depth, notably its impact on mother's immunity and its consequence on offspring protection.
6. Immune status and fitness costs of offspring from challenged parents should be investigated at different developmental stages to account for potential stage-specificity of TGIP and avoid missing its expression. Moreover, different mechanisms might be at play at the different developmental stages and investigating only a limited number of stages could bias the analysis of TGIP process. The age of the mother (and potentially the father too) should also be monitored considering that older females might not invest as much in offspring protection as younger ones.
7. Several successive generations should be monitored to see if TGIP is a sustained process or if it is restricted to the first generation, which could help in deciphering the underlying mechanisms, notably epigenetic ones.
8. The role of microbiota in TGIP must be specifically investigated. Its ability to modulate the parents'—and potentially offspring's—innate immune response can strongly affect the outcome of TGIP and bias our understanding of the phenomenon at both the epidemiologic and mechanistic level.
9. Last but not least, all articles investigating TGIP mechanisms by the means of molecular approaches such as transcriptomic, proteomic or enzymatic activities (TEI) should also systematically monitor the enhanced offspring resistance (TER), notably by measuring the offspring survival to the studied pathogen(s) and the parasite load. This is mandatory to be able to properly compare different studies and to decipher all the complexity of trans-generational immune priming because, as Tom J. Little and collaborators wrote in 2005, “*without analogous experiments, mechanism-driven work may not demonstrate the full richness of invertebrate immunity*” (153).

## Author Contributions

GT initiated the project. All authors participated to the discussions and writing of the article.

### Conflict of Interest Statement

The authors declare that the research was conducted in the absence of any commercial or financial relationships that could be construed as a potential conflict of interest.

## Supplementary Material

Supplementary Table 1 List of the 57 articles investigating TGIP in invertebrates. This table contains details about the host/pathogens studied, the experimental procedure used and the outcome of the experiments.
